# Profiling the airway in the macaque model of tuberculosis reveals variable microbial dysbiosis and alteration of community structure

**DOI:** 10.1186/s40168-018-0560-y

**Published:** 2018-10-09

**Authors:** Anthony M Cadena, Yixuan Ma, Tao Ding, MacKenzie Bryant, Pauline Maiello, Adam Geber, Philana Ling Lin, JoAnne L Flynn, Elodie Ghedin

**Affiliations:** 1 Department of Microbiology and Molecular Genetics, University of Pittsburgh School of Medicine, Pittsburgh, PA USA; 2 Present address: Center for Virology and Vaccine Research, Beth Israel Deaconess Medical Center, Harvard Medical School, Boston, MA USA; 3 Center for Genomics and Systems Biology, Department of Biology, New York University, New York, USA; 4 Present address: Department of Pediatrics, University of California, San Diego School of Medicine, La Jolla, California, USA; 5 Department of Pediatrics, Children’s Hospital of Pittsburgh of University of Pittsburgh Medical Center, University of Pittsburgh School of Medicine, Pittsburgh, PA USA; 6 College of Global Public Health, New York University, New York, NY USA

**Keywords:** Mycobacterium tuberculosis, Lung and airway microbiota, Microbiome, 16S rRNA gene, Cynomolgus macaque

## Abstract

**Background:**

The specific interactions of *Mycobacterium tuberculosis* (Mtb), the causative agent of tuberculosis (TB), and the lung microbiota in infection are entirely unexplored. Studies in cancer and other infectious diseases suggest that there are important exchanges occurring between host and microbiota that influence the immunological landscape. This can result in alterations in immune regulation and inflammation both locally and systemically. To assess whether Mtb infection modifies the lung microbiome, and identify changes in microbial abundance and diversity as a function of pulmonary inflammation, we compared infected and uninfected lung lobe washes collected serially from 26 macaques by bronchoalveolar lavage over the course of infection.

**Results:**

We found that Mtb induced an initial increase in lung microbial diversity at 1 month post infection that normalized by 5 months of infection across all macaques. Several core genera showed global shifts from baseline and throughout infection. Moreover, we identified several specific taxa normally associated with the oral microbiome that increased in relative abundance in the lung following Mtb infection, including *SR1*, *Aggregatibacter*, *Leptotrichia*, *Prevotella*, and *Campylobacter*. On an individual macaque level, we found significant heterogeneity in both the magnitude and duration of change within the lung microbial community that was unrelated to lung inflammation and lobe involvement as seen by positron emission tomography/computed tomography (PET/CT) imaging. By comparing microbial interaction networks pre- and post-infection using the predictive algorithm SPIEC-EASI, we observe that extra connections are gained by *Actinomycetales*, the order containing Mtb, in spite of an overall reduction in the number of interactions of the whole community post-infection, implicating Mtb-driven ecological reorganization within the lung.

**Conclusions:**

This study is the first to probe the dynamic interplay between Mtb and host microbiota longitudinally and in the macaque lung. Our findings suggest that Mtb can alter the microbial landscape of infected lung lobes and that these interactions induce dysbiosis that can disrupt oral-airway boundaries, shift overall lung diversity, and modulate specific microbial relationships. We also provide evidence that this effect is heterogeneous across different macaques. Overall, however, the changes to the airway microbiota after Mtb infection were surprisingly modest, despite a range of Mtb-induced pulmonary inflammation in this cohort of macaques.

**Electronic supplementary material:**

The online version of this article (10.1186/s40168-018-0560-y) contains supplementary material, which is available to authorized users.

## Background

Microbial dysbiosis is increasingly recognized as a significant factor influencing human disease and host immunity in a variety of contexts spanning allergy, autoimmunity, cancer, and infectious disease [1–6]. In the context of infectious disease, studies have shown that the microbiome has important influences on the immunological landscape by altering immune regulation, inflammation, and pathogen colonization [2, 7–9]. The gut has been the primary focus of a number of studies linking the immune system and an organ’s microbiome [3, 10, 11], but it is increasingly clear that other human immune surfaces and interfaces, including the lung, are actively shaped by host-microbe interactions [12–15]. How specific pathogens modify and influence host microbial landscapes and site-specific immunity, particularly in the lung, is poorly understood. In particular, very little is known about the interaction of the lung microbiome and *Mycobacterium tuberculosis* (Mtb), the bacillus that causes tuberculosis (TB). Human Mtb infection presents along a spectrum of host outcomes with only ~ 10% presenting with active TB, a clinically defined state of infection that includes chronic cough, hemoptysis, weight loss, night sweats, and fever, and may manifest for years until clinically diagnosed [16–18]. By contrast, the majority of Mtb infections (90%) are successfully contained in an asymptomatic state termed latent TB infection (LTBI) that can persist for their lifetime [16, 18, 19]. Current estimates place 2 billion humans within this clinically latent reservoir [19, 20] who are at risk of reactivation and progression to active TB. The precise mechanisms driving the variability in human Mtb infection outcome are poorly understood but may include the earliest interactions between pathogen and the local lung microbiota [21].

Previous work in human TB and the lung microbiome suggested that TB patients have altered diversity in their airway microbiota compared to uninfected humans [22–24] but these studies have been limited by their cross-sectional nature and some are confined to the sputum. The distribution of some genera (*Stenotrophomonas*, *Phenylobacterium*, *and Cupriavidus)* was unique to TB patients [23, 24]. Certain patterns of microbiota (e.g., presence of *Pseudomonas spp.*) were observed in patients with recurrent TB and treatment failure, suggesting that the lung microbiota interacts with both host and pathogen [25]. Specifically, alterations in *Treponema* and *Atopobium* were associated with recurrent TB suggesting that an altered “normal” microbiome correlated with ongoing susceptibility to TB [25]. Studies examining the microbiota of the oropharynx between healthy controls and TB patients showed differences in diversity and abundance of particular organisms although no differences in major phyla were observed [26]. Other studies in experimental murine models of infection have noted distinct changes in the gut microbiome following Mtb challenge [27], although the precise effects and relationship of these changes on course of infection were not explored. A more recent study profiled alterations in the gut microbiota after tuberculosis antibiotic treatment in mice and observed a distinct change in bacterial taxa that persisted months after cessation of treatment [28]. Collectively, this body of work hints at a related immune—lung microbiome axis that extends from the lung to the oropharynx, as well as the gut, that exhibits significant cross-talk following Mtb infection.

Here, using a macaque model of TB, we explored for the first time whether Mtb infection alters the lung microbiota in a significant and durable manner, and whether the microbial shift is associated with pulmonary inflammation. By serially sampling the airway of 26 infected macaques via bronchoalveolar lavage (BAL), we have provided the first survey of the microbial lung landscape over time in the context of this chronic lung pathogen.

## Results

### Lung microbial richness and composition shift during Mtb infection

To determine whether infection with Mtb had an effect on the microbial composition of the macaque lung, we sampled 26 macaques at baseline (2 weeks pre-infection) and at 3 time points post-infection (months 1, 4, and 5 post-infection) (Table 1). For each time point, a bronchoalveolar lavage (BAL) sample was collected from the right and left lobes, separately. As a negative control, a saline wash sample of the bronchoscope was also obtained. To confirm that the BAL sampling would specifically access the lungs and not be biased by the microbial community of the oral cavity, we also collected oral wash from each animal. We determined by statistical testing that the microbial communities of these two compartments were significantly different (*p* < 0.001), as visualized by t-Distributed Stochastic Neighbor Embedding (t-SNE) analysis (Additional file 1: Figure S1).

**Table 1:** Number of samples for each sample type, including controls

Cohort	Macaques	Oral washes	Bronchoscope controls	BAL of lobes	Reagent control	Total number
1	10	36	36	70	20	162
2	16	55	55	108	32	250
Total	26	91	91	178	52	412

**Fig. 1:**
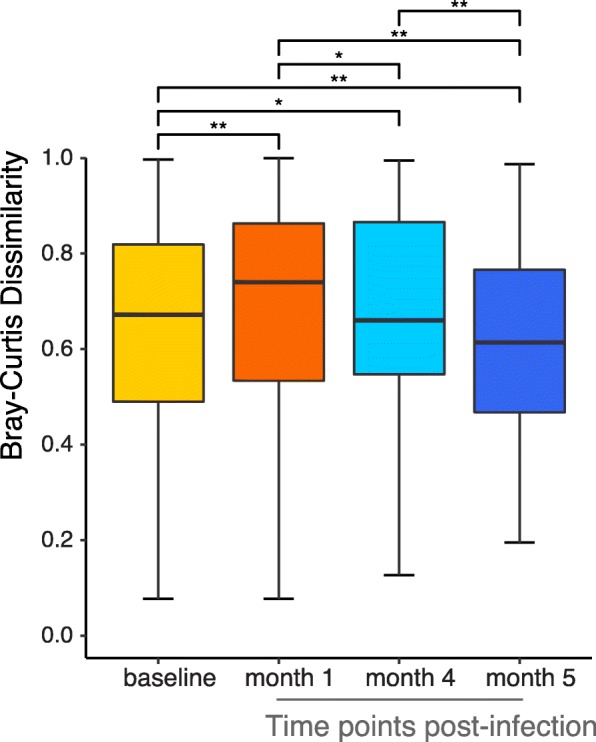
Beta-diversity for the lung microbiota of Mtb infection. Boxplots of the beta diversity (calculated as Bray-Curtis dissimilarity) between BAL samples grouped by time point. *Y* axis: Bray-Curtis distance. Whiskers represent values outside the upper and lower quartiles. *p value between 0.01 and 0.05; **p value < 0.001

We compared the microbial communities between samples (Beta-diversity) at baseline and post-infection using Bray-Curtis distance as a measure of similarity (the more similar the communities, the shorter the distance). Visualized by t-SNE, there appears to be no clustering of the samples based on their infection status (baseline versus post-infection) or by specific time point (Additional file 2: Figure S2). However, when we performed a Wilcoxon test, we observed a small but significant change post-infection compared to the baseline, with an initial overall increase in Beta-diversity at 1 month followed by a decline in Beta-diversity at months 4 and 5 (Fig. 1), indicating that microbial communities of the samples are becoming more similar at these later time points.

**Fig. 2:**
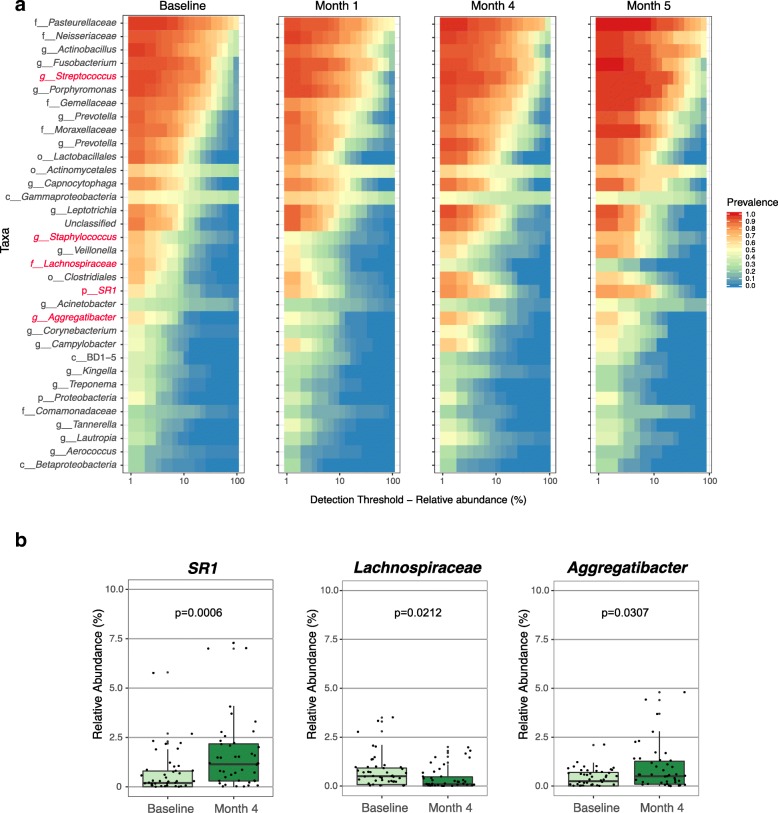
**a** Core microbiome heatmaps showing abundance of taxa and prevalence across samples at baseline and at each time point, providing a measure of the lung microbiota dynamics in the progression of Mtb infection. Highlighted in red are some of the taxa that change significantly over the course of the infection. Letters appended before names indicate whether the taxonomic assignment was made at the phylum (p_), class (c_), order (o_), family (f_), or genus (g_) level. **b** Boxplots of relative abundance of significant taxa enriched in the lung airways at baseline and at month 4. Significance was determined by LefSE. Whiskers indicate the highest or lowest occurring value within 1.5*IQR (interquartile range) of the upper or lower quartile

When performing analyses of the microbial communities at the level of single macaques, we see unique compositions of bacterial taxa that shifted over the course of the infection (Additional file 3: Figure S3). Although the macaques were given the same food, they were single-housed, thus the variability observed in the respiratory microbiota is not surprising given the genetic variability of macaques and the different exposures of each monkey over time, including antibiotic treatment (prior to Mtb infection) as well as the host variability in TB [18]. To identify global shifts in microbial composition across all macaques, we focused on the core microbiota, which is comprised of taxa with a minimum relative abundance of 0.1% that are shared by 95% of the subjects. We thus identified four core microbiota, one for each time point, and we visualized the common taxa across these time points (Fig. 2a). We compared baseline samples with post-infection samples at each time point by Linear discriminant analysis Effect Size (LEfSe). We identified three taxa that shifted significantly between baseline and month 4, a time point at which host outcome is well established [21, 29]. *Lachnospiraceae* (*p* value = 0.0212) was present at a higher relative abundance in the pre-infection compared with post-infection samples at month 4, whereas *SR1* (*p* value = 0.0006) and *Aggregatibacter* (*p* value = 0.0307) were enriched post-infection at month 4 (Fig. 2b). The family *Lachnospiraceae* and the unculturable Candidate division SR1 bacteria have previously been found in the mammalian gut [30] and mouth [31, 32], respectively, and an oral strain of *Aggregibacter* was sequenced from the Rhesus macaque [33], indicating that these are likely commensal bacteria that have shifted in relative abundance with Mtb infection. Comparisons of core microbiota across other time points also led to the identification of taxa such as *Staphylococcus* and *Streptococcus* from month 1 to month 4 (*p* = 0.049 and *p* = 0.040, respectively).

### Community abundance and structure are not associated with pulmonary inflammation or lobe involvement in TB

**Fig. 3:**
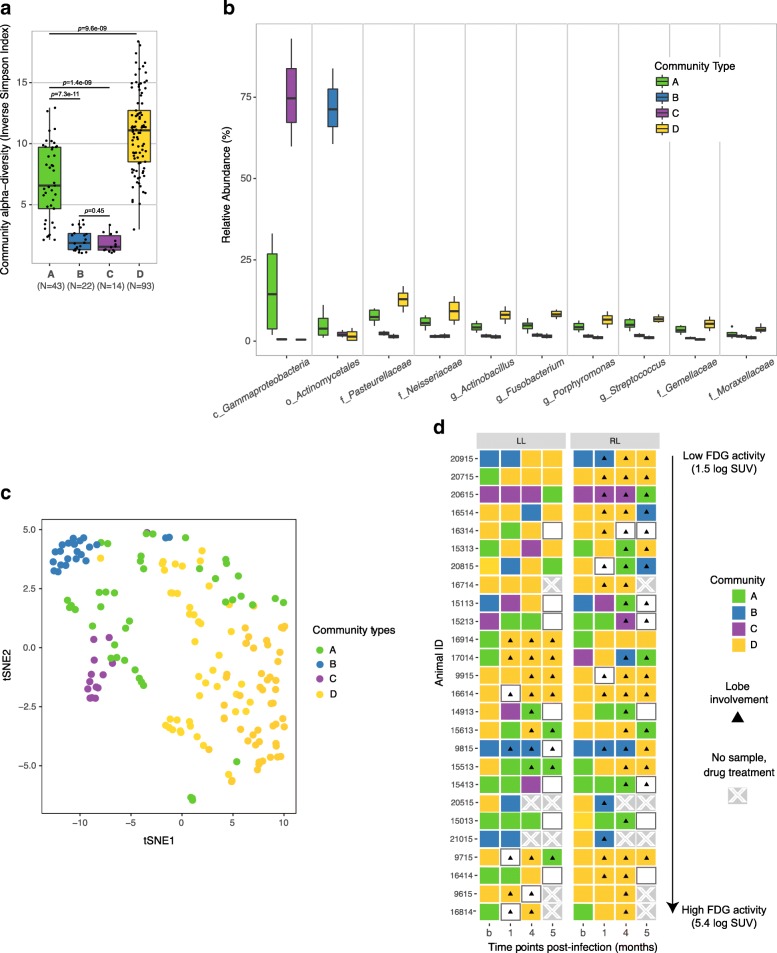
Community types found in the lung microbiota. **a** Alpha diversity. *p* values from Wilcoxon rank sum tests are included. The alpha diversities of community types B and C were overall significantly lower (*p* value = 2.2e-16) than those of community types A and D. **b** Relative abundance of taxa over-represented in each community type. Letters appended before names indicate whether the taxonomic assignment was made at the class (c_), order (o_), family (f_), or genus (g_) level. **c** tSNE, colored by community types. **d** Community type that predominates in each monkey at each time point. Triangles represent radiographic evidence of lung pathology (i.e., a granuloma or other grossly visible disease at a resolution of 1 mm by CT) that coincide with microbiome sampling at the indicated time point. From top to bottom (arrow), monkeys were ordered, low to high, by the log transformed total [^18^F]-FDG activity at 4–5 months. Xs indicate samples not collected; white (blank) boxes indicate that the sequence depth was not sufficient for analysis (fewer than 1000 sequence reads) and so the sample was not included

To investigate whether the dynamics of the microbiota are associated with progression of disease and its accompanying inflammation in the macaques, we performed a multivariate statistical analysis that determines associations between clinical metadata and microbial community abundance (MaAsLin). We did not identify any specific taxa that were specifically correlated with pulmonary inflammation throughout the course of infection in these animals (data not shown). We further explored correlations with inflammation and disease involvement by looking at the community structure as a whole. To do that, we partitioned the data into community types using Dirichlet multinomial mixture models [34]. We identified four major community types in the lung microbiota. Community types B and C had the lowest alpha-diversities (Fig. 3a) and were dominated by one taxon each: *Gammaproteobacteria* and *Actinomycetales*, respectively (Fig. 3b). Community types A and D were each represented by a mix of taxa at different relative abundances. *Capnocytophaga* was over-represented in community type D but at a relative abundance between 2 and 3% (not shown). When labeling the samples by their community types and visualizing their beta-diversity, we see that communities B and C stand out as the tightest clusters (Fig. 3c). To determine the stability of the community types over the course of the infection, we mapped the communities at each time point across the lung samples and in each monkey (Fig. 3d). Monkeys were rank ordered by their inflammation status (least to greatest), as measured by their total lung [^18^F]fluoro-D-glucose (FDG) uptake by positron emission tomography and computed tomography (PET/CT) scans at 4–5 months post infection (Table 2). We previously published that total lung FDG activity correlates with thoracic bacterial burden in macaques [35]. Lung side involvement was determined by visual confirmation of disease pathology by PET/CT scan at the respective time point post-infection. We observed several community shifts during infection in a number of monkeys, but the microbial community appears stable in others. Community types B and C were over-represented in samples that come largely from two monkeys (IDs 20615 and 9815). Community type D was the most prominent community found throughout infection, and between left and right lung lobes. There was no discernible relationship between the community types or shift in community type over time and inflammation status or lung side involvement (Fig. 3d).

**Table 2:** [^18^F]-FDG uptake values for each monkey at 4–5 months post infection by PET/CT

Macaques	PET HOT (4–5 months (SUV)	Log_10_(PET HOT 4–5 months)
20,915	36.27	1.56
20,715	655.30	2.82
20,615	903.38	2.96
16,514	911.11	2.96
16,314	1102.36	3.04
15,314	1320.82	3.12
20,815	1426.94	3.15
16,714	3498.34	3.54
15,113	3701.98	3.57
15,213	5264.63	3.72
16,914	5724.37	3.76
17,014	6492.61	3.81
9915	7166.13	3.86
16,614	7832.35	3.89
14,913	8517.50	3.93
15,613	10,563.85	4.02
9815	14,896.56	4.17
15,513	18,738.09	4.27
15,413	27,612.06	4.44
20,515	30,032.82	4.48
15,013	33,494.44	4.52
21,015	39,013.51	4.59
9715	53,815.37	4.73
16,414	121,015.76	5.08
16,814	277,171.63	5.44

### Infection with Mtb leads to modified microbial interactions

**Fig. 4:**
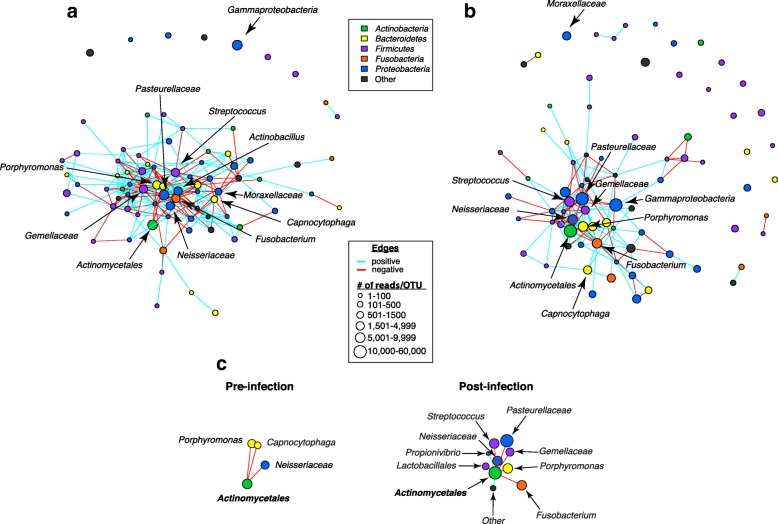
Microbial co-occurrence networks determined with SPIEC-EASI. Mtb infection modulates microbial interactions selecting for both positive (blue edges) and negative (red edges) interactions between taxa. **a** Correlation network for pre-infection samples. **b** Correlation network for post-infection samples. **c** Nodes and edges connecting *Actinomycetales* with its partners pre- and post-infection. Each node represents a taxon and is colored by its assigned phylum. Blue edges represent positive correlation between taxa and red represents negative correlation. Node area corresponds to OTU abundance; the sizes of the nodes were scaled by the number of reads for each OTU. Reads of OTUs were grouped into six ranges: 1–100, 101–500, 501–1500, 1501–4999, 5001–9999, 10,000–60,000

To determine how specific microbes potentially interact with each other in the community and how their interactions are disrupted by Mtb infection, we compared co-occurrence of taxa over the course of the Mtb infection using the SPIEC-EASI algorithm (Fig. 4), which infers a graphical model using conditional independence between OTUs [36, 37]. In general, we see that the total number of microbial interactions, as indicated by the number of edges between the nodes, decreased post-infection (pre: *n* = 122, post: *n* = 104) (Fig. 4 and Table 3). Of the taxa profiled, *Porphyromonas*, *Capnocytophaga*, and *Moraxellaceae* had the greatest loss in predicted interactions with the latter losing all of its interactions indicating it has become conditionally independent of other taxa present. By contrast, *Actinomycetales*—the order that includes mycobacteria—which only had 3 connections (all positive) in pre-infection samples, had 9 connections in post-infection samples, including 2 positive and 7 negative correlations. This implies that Mtb affects the overall network and competes with specific taxa. We also observed that the diverse class *Gammaproteobacteria*, which contains a number of important human pathogens including *Pseudomonas*, *Yersinia*, *Salmonella*, *Vibrio*, and *Escherichia coli* [38] established six new taxonomic interactions in the airway and became a central node following Mtb infection.

**Table 3:** Summary of edges (positive and negative) for each taxon

	Pre-infection			Post-infection		
	Total	Positive	Negative	Total	Positive	Negative
*Pasteurellaceae*	18	9	9	17	7	10
*Neisseriaceae*	12	9	3	9	6	3
*Actinobacillus*	13	9	4	8	6	2
Actinomycetales*	3	0	3	9	2	**7**
*Gammaproteobacteria*	0	–	–	6	2	4
*Fusobacterium*	18	7	11	15	8	7
*Streptococcus*	14	6	8	15	5	10
*Porphyromonas*	18	10	8	11	8	3
*Moraxellaceae*	8	2	6	0	–	–
*Gemellaceae*	17	9	8	10	5	5
*Capnocytophaga*	11	6	5	4	4	0

*Order to which Mtb belongs

## Discussion

In this study, we coupled our cynomolgus macaque model of TB [35, 39] with the ability to serially sample the airways of animals throughout the course of lung infection for microbiome analysis. We exploited the [^18^F]-FDG PET/CT imaging analyses that we have developed for use in this model to assess infection trajectory and severity combined with microbial profiling [29, 40–45] to determine whether Mtb infection changed the lung microbial community and the duration of that effect. As a result, our study is the first targeted exploration of the lung microbiome in an animal model that faithfully replicates human Mtb infection outcomes and pathology. Leveraging these imaging and sequencing technologies with our TB animal model, we found that Mtb induces an initial shift in the overall microbial richness that peaks at 1 month post infection and returns to baseline by 5 months across our infected macaques (Fig. 1); there was no discernible clustering that occurred between pre-infection and any time points post-infection of our monkeys in aggregate (Additional file 2: Figure S2). Importantly, we observed a high degree of variability in airway microbial change across the different macaques: several monkeys displayed robust microbial shifts while other monkeys harbored more stable lung communities (Additional file 3: Figure S3) likely reflecting the outbred nature of the animals, variable pre-Mtb exposures and treatments, and the heterogeneity in Mtb infections in macaques [18]. We postulate that this observed microbial heterogeneity may in turn contribute to the spectrum of host outcome seen in TB by modifying initial airway interactions between host cells and Mtb or specific granuloma microenvironments.

When we profiled core taxa for specific changes in relative abundance and prevalence following Mtb infection, we found several shifts of residents associated with the mammalian oral microbiota into the airway. These included members of the *Aggregatibacter*, *Streptococcus*, and *Staphylococcus* (Fig. 2) genera. Their enrichment over the course of Mtb infection hints at increased transit of oral microbes into the lower airway. While LEfSe analysis identified both *SR1* and *Aggregatibacter* as significantly enriched at 4 months post challenge, *Lachnospiracea*, a family associated with the intestinal microbiome [46] and recently linked to adiposity following broad spectrum antibiotic challenge [30], was significantly decreased.

To further address functional microbe relationships within the airways, we profiled microbe interaction networks at both pre- and post-infection using SPIEC-EASI [37]. We saw that the majority of taxa have a net loss of their total predicted interactions (both positive and negative) post Mtb challenge; by contrast, *Actinomycetales*, the order that contains Mtb, had an overall increase in negative correlations. Other notable network shifts included the transition of *Gammaproteobacteria* from a peripheral node to a central node with the predicted increase of six new edges following Mtb challenge. Collectively, these microbial correlation networks imply that Mtb may alter the functional interactions of microbes residing within the airways during infection, possibly to better establish a niche for infection. It posits unique microbe-microbe relationships secondary to infection that may initiate local dysbiosis and potentially influence barrier integrity, lung immunity, and host inflammation [13, 14, 47].

While relating specific host disease status and TB outcome with alteration of lung microbiota was beyond the scope of this pilot study (profiled monkeys were pooled from multiple studies with differing infection lengths and experimental endpoints), we observed remarkable stability in the overall lung microbiota post Mtb challenge. This relative stability was evident in both involved and uninvolved lung lobes (lobes with and without visible disease) and in lung lobes with both high and low levels of pulmonary inflammation (Fig. 3). Although this stability was not entirely universal, as was highlighted above with some macaques exhibiting greater shifts in both relative abundance and community structure, it is surprising that a robust lung bacterial infection did not cause a more significant dysbiosis of the airways. Interestingly, Morris and colleagues [48] also reported an absence of systemic or persistent alteration of the macaque lung microbiota following long-term SHIV infection despite notable host-specific variation and concomitant immunosuppression. Similar non-discriminatory results were seen in a recent study of the oral and lung microbiota of HIV-infected individuals (treatment naïve median CD4 count = 668 cells/mm^3^ and HAART median CD4 count = 618 cells/mm^3^) compared against HIV-negative controls [49]. A separate lung study of HIV+ individuals with more advanced disease (baseline mean CD4 count = 262 cells/mm^3^) did, however, find significant alterations in both bacterial diversity and composition compared to an uninfected population [50]. The observations from the human studies, the macaque SHIV model, and the findings in our Mtb-infected cynomolgus macaques suggest that the bacterial landscape within the airway is a highly variable mucosal surface with indications of both resistance and plasticity that is influenced by the type of pathogen, the initial status of the lung, the severity of disease, host inflammation, and the incidence of exacerbation [13, 14, 47]. However, there are key features of tuberculosis that may contribute to the relatively modest overall changes in airway microbiota we observed following Mtb infection. First, Mtb is a relatively slowly evolving infection, since the bacteria replicate quite slowly in vitro and in vivo; our previous studies suggested a doubling time in macaque lung of ~ 40 h in the first month of infection [51]. Acute lung infections could have a stronger impact on airway microbiome composition [14, 52]. Second, TB is primarily a parenchymal infection rather than a strict airway infection, so there may be stronger effects on the actual lung vs. the airway microbiota. It is possible that much more severe TB disease, including cavitary disease, would have a larger impact on the airway microbiome. Third, Mtb infection results in the formation of granulomas, which are designed to compartmentalize the infection, protecting the lung from further infection spread, and thus effects on microbiome may be more local. Finally, unlike the gut, the lungs and airways are sparsely populated by microbes, and there may be adequate space for new microbial agents to enter and replicate without major changes to the microbial residents. A better understanding of the interaction of the lung microbiota, TB disease pathogenesis, and host response will rely on a more targeted exploration of the local, granuloma microenvironment [18, 53, 54], as well as sequencing approaches including transcriptomics and metagenomics that complement and enrich 16S rRNA gene sequence analyses.

## Conclusions

By serially sampling the BAL of both infected and uninfected lung lobes in macaques over the course of Mtb infection, we have provided an initial survey into how Mtb modifies the microbial environment of the lung. We observed alteration of the lung microbiota post challenge in early infection as well as significant intra-host variation that reflects the spectrum of outcomes observed in TB. We identified several changes among the microbial interactome that posit an Mtb-mediated reshuffling of intra-microbial relationships. However, despite the presence of a wide range of pulmonary inflammation in the macaques studied here, there was a relatively modest effect overall on the airway microbiome in response to Mtb infection, and the effects observed were quite variable among subjects.

## Methods

### Macaque infections and imaging, microbiome experimental design, and sample collection

In this study, we analyzed oral washes (OW), bronchoalveolar lavages (BALs), and bronchoscope control samples of 26 cynomolgus macaques (*Macacca fasicularis*) obtained from Valley Biosystems (Sacramento, CA). All animals underwent baseline blood and chemical profiles and were housed in biosafety level 3 facilities in accordance with the standards of the Animal Welfare Act and the *Guide for the Care and Use of Laboratory Animals.* All macaque procedures and protocols were approved by the University of Pittsburgh’s Institutional Animal Care and Use Committee.

The macaques were infected with < 25 CFU Mtb strain Erdman via bronchoscopic instillation into the right lower lung, as previously described [39]. A 5 mL oral wash was first obtained from the cheek pouch. A 5 mL saline wash of the sterilized bronchoscope was also obtained to detect any residual DNA within the scope. The mouth was swabbed with an antiseptic agent (chlorohexane) immediately prior to endotracheal insertion of the bronchoscope. Saline lavage (7 mL each lobe) of the right and left lower lung lobes was performed with scope sterilization between entering each lobe. On average, 4–5 mL were obtained from both sides of the lung (left and right lobes) for each macaque at 2 weeks before infection (considered the baseline), and 1, 4, and 5 months post-infection. The samples were stored at − 80 °C until they were processed (Table 1). Macaques were serially imaged throughout infection with 2-deoxy-2-[^18^F]fluoro-D-glucose positron emission tomography and computed tomography ([^18^F]-FDG PET/CT), as previously published [29, 54, 55], to track inflammation and disease progression. Total lung inflammation (total FDG activity) is a cumulative summation of [^18^F]-FDG uptake (as measured by standard uptake value [SUV] with positive PET above 2.3) [35, 56](Table 2).

### DNA isolation, amplification, and sequencing

DNA was isolated from 5 mL of the oral wash, 5 mL of the bronchoscope wash, and 4–5 mL lavage from each lobe using the PowerSoil® DNA Isolation Kit (MO BIO Laboratories Inc.). DNA isolations were performed at the University of Pittsburgh in a dedicated biosafety level 3 cabinet that was first exposed to a UV light source for 15 min. Reagent and sterile PBS were also processed through the DNA isolation procedure as negative controls. The DNA was eluted in 100 μL elution buffer. Each sample was quantified with the Qubit 2.0 Fluorometer (ThermoFisher Scientific Inc.). The samples were stored at − 20 °C until shipment to New York University for analysis.

Extracted DNA was used in a PCR reaction with sequencing primers targeting the V4 hypervariable region of the bacterial 16S ribosomal RNA (rRNA) gene. 6 μL of DNA were amplified in a final volume of 25 μL with 0.35 μL Q5 Hot Start High-Fidelity DNA Polymerase (New England BioLabs Inc.), 5 μL 5X Q5 Buffer, 0.5 μL dNTP and 5 μM primers (515F/806R) [57], 5 μM barcoded reverse primer and water. Cycling conditions were 94 °C for 2 min, then 33 cycles at 94 °C for 30 s, 55 °C for 30 s, and 72 °C for 30 s, followed by 72 °C for 10 min.

Reactions were cleaned up using 0.65× volume of AMPure XP Beads (Agencourt) and eluted into 20 μL low TE, pH 8.0 on the Bravo Automated Liquid Handling Platform (Agilent Technologies). Eluted PCR products were quantified with a Quant-iT double-stranded DNA (ds-DNA) High-Sensitivity Assay Kit (Invitrogen) according to the manufacturer’s instructions and combined into a pool with equal amounts of each amplicon. Samples were then pooled and each pool was re-purified with 0.65× volume of AMPure XP Beads (Agencourt). All pools were re-run on a HSD1000 ScreenTape with the TapeStation instrument (Agilent Technologies) and quantified with the Qubit 2.0 Fluorometer. Three pools were combined into a final pool with equal amounts of each amplicon and re-purified with 0.65× volume of AMPure XP Beads to ensure library quality. The pool was quantified by qPCR with the KAPA Library Quantification Kit (KAPA Biosystems) for the Roche 480 LightCycler system. The final library was 2 × 250 bp paired-end sequenced on the MiSeq Illumina Sequencer at the Genomics Core Facility of the Center for Genomics and Systems Biology (CGSB), New York University.

### Sequence data processing and analysis

The 16S rRNA gene sequences were processed using the Quantitative Insights into Microbial Ecology (QIIME) pipeline for analysis of microbiome data [58]. The reads were end-paired with the 1.1.2 *ea-utils* [59], filtered, and de-multiplexed. Chimeric sequences were identified and filtered out with the ChimeraSlayer sequence detection tool [60]. Sequences were clustered into Operational Taxonomic Units (OTUs) at a 97% similarity threshold with the open-reference approach, which begins by running a close-reference step followed by a de novo step that clustered the sequences that failed closed-reference assignment. Taxonomic assignments of the sequences were made using the RDP (Ribosomal Database Project) classifier [61], which classifies sequences to the species level, aligning the reference sequences picked for each OTU against the 16S rRNA gene database Greengenes 13_5 (version of May 2013) [62]. Assignments of taxonomy were refined though phylogenetic methods using PyNAST [63] to generate an alignment for each cluster from which a phylogenetic tree was generated using the FastTree approximately maximal likelihood method [64]. We identified *Burkholderia* as being one of the dominant genera in the bronchoscope controls, and so any OTU matching that genus was removed from the analyses. After removal of *Burkholderia*, we identified 16,218 OTUs (from an original total of 16,243) with a mean of 26,600 reads per sample and 511 genus-level OTUs. Singletons and OTUs present in less than 10% of the samples were filtered out of the dataset to reduce statistical noise, leaving the 98 most abundant OTUs for downstream analyses. Samples with fewer than 1000 reads were removed, and all others were subsampled/rarified to 1000 reads.

The QIIME output data were imported in RStudio (Version 1.0.136) with the Bioconductor package phyloseq [65], including subsetting, normalizing, and plotting of the input data. The taxonomic and OTU-based profiles were used in a series of ordination, clustering, and community diversity analyses designed to identify significant shifts in 16S rRNA gene profiles between samples across time points and for further statistical analyses. Alpha diversities of different groups, calculated as the inverse Simpson Index, were compared using the Wilcoxon signed-rank test. The beta-diversity was calculated as Bray-Curtis dissimilarity [66] and analyzed/compared using t-Distributed Stochastic Neighbor Embedding (t-SNE) [67] which allows dimensionality reduction.

For OTU differential relative abundance analysis, the Linear Discriminant Analysis Effect Size (LEfSe) [68] method was applied. This method consists of a Kruskal-Wallis test followed by subsequent Wilcoxon rank-sum tests on subgroups. This analysis was performed using the Huttenhower lab online Galaxy web application [69]. For the association analysis between microbial abundance and lung inflammation, Multivariate Association with Linear Models (MaAsLin) analysis was applied [70]. This analysis was performed using the MaAsLin R package (https://bitbucket.org/biobakery/maaslin/downloads/Maaslin_0.0.4.tar.gz).

Ecological correlation networks were constructed to identify interactions of the microbial community associated with Mtb infection. Taxa counts were normalized using total sum scaling (also known as relative abundance) followed by centered log ratio scaling [71]. Each network was built using SParse InversE Covariance estimation for Ecological ASsociation Inference (SPIEC-EASI) package version 0.1 in R (https://github.com/zdk123/SpiecEasi) [37, 36]. The sparse graphical lasso (glasso) setting was used and the optimal sparsity parameter was selected, based on the Stability Approach to Regularization Selection (StARS) [72]. The StARS variability threshold was set to 0.1 for all networks. Networks were analyzed using functions of the R package igraph version 1.0.1 [73].

Community types [74] of the lung microbiome were identified using the Dirichlet Multinomial Mixture (DMM) model [34], implemented in mothur [75]. The Laplace approximation was used for selecting the best number of community types, and the optimal number of community types was found when the minimum Laplace value was observed.

## Additional files

Additional file 1: **Figure S1.** tSNE showing clustering of oral and BAL samples. Yellow: oral wash (OW); Red: bronchoalveolar lavage samples (BAL); triangles: pre-infection samples: circles: post-infection samples. (PDF 132 kb)

Additional file 2: **Figure S2.** tSNE of the lung microbiota, measured by Bray-Curtis distances. The different colors correspond to the different time points. Yellow: baseline, *n* = 52 samples; orange: month 1, *n* = 47; light blue: month 4, *n* = 46; dark blue: month 5, *n* = 27. (PDF 702 kb)

Additional file 3: **Figure S3.** Relative abundance of main taxa in the lung microbiota. For most monkeys, the microbial composition is relatively similar between right and left lobes within the same monkey, while the microbiota across monkeys is divergent. b: baseline; 1, 4, 5: months post-infection. LL: left lower lobe; RL: right lower lobe. (PDF 287 kb)

## Abbreviations

[^18^F]-FDG: 2-deoxy-2-[^18^F]fluoro-D-glucose
16S rRNA gene: 16S subunit of the ribosomal RNA gene
BAL: Bronchoalveolar lavage
CT: Computed tomography
DMM: Dirichlet multinomial mixture
LEfSe: Linear discriminant analysis effect size
MaAsLin: Multivariate Association with Linear Models
Mtb: *Mycobacterium tuberculosis*
OTU: Operational taxonomic unit
PET: Positron emission tomography
QIIME: Quantitative Insights into Microbial Ecology
SPIEC-EASI: Sparse InversE Covariance estimation for Ecological ASociation Inference (pronounced as “speak easy”)
StARS: Stability Approach to Regularization Selection
TB: Tuberculosis
t-SNE: t-Distributed Stochastic Neighbor Embedding
