# Supplementary figures and images for: Profiling the airway in the macaque model of tuberculosis reveals variable microbial dysbiosis and alteration of community structure
Source: Microbiome. 2018 Oct 9;6:180. doi: 10.1186/s40168-018-0560-y (PMC6178261; doi:10.1186/s40168-018-0560-y)

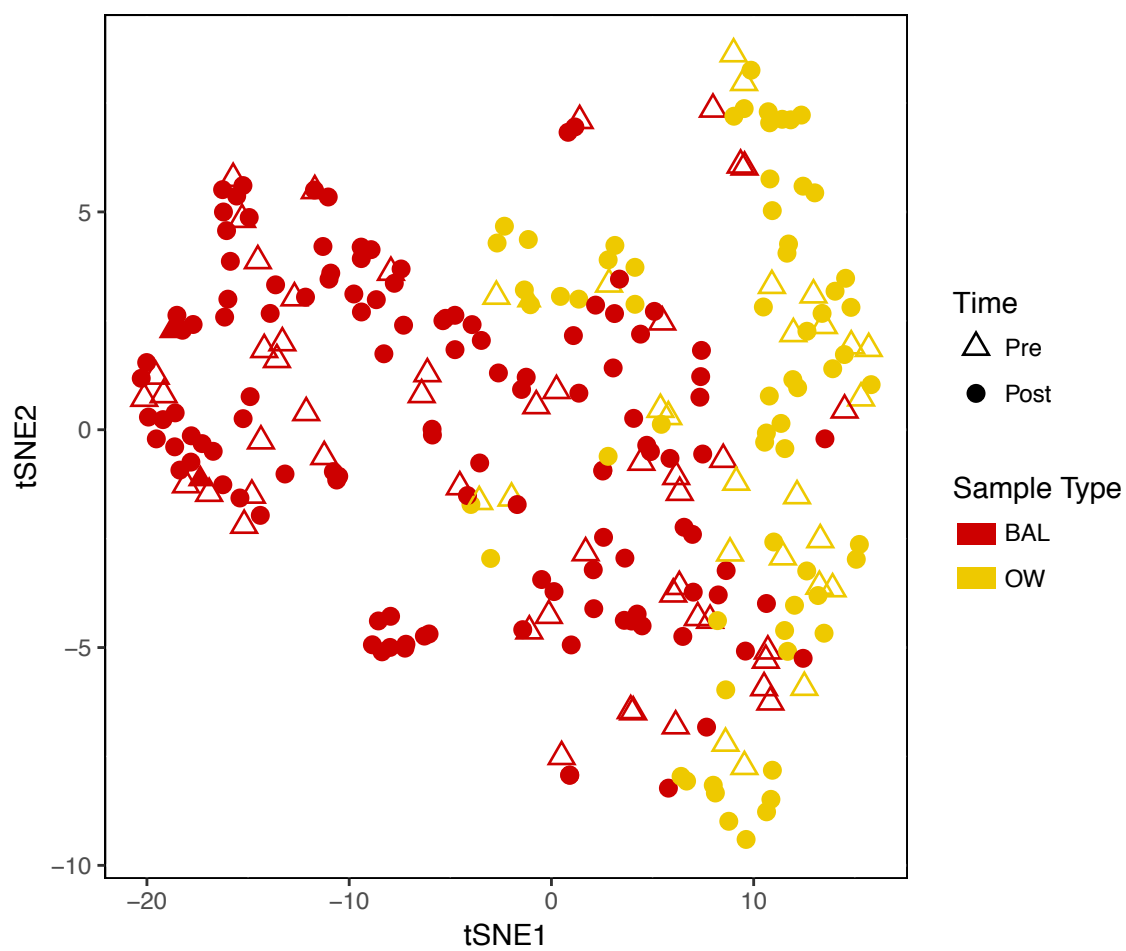

Supplement: Supplementary file 1 — Figure S1. tSNE showing clustering of oral and BAL samples. Yellow: oral wash (OW); Red: bronchoalveolar lavage samples (BAL); triangles: pre-infection samples: circles: post-infection samples. (PDF 132 kb) [file 40168_2018_560_MOESM1_ESM.pdf]

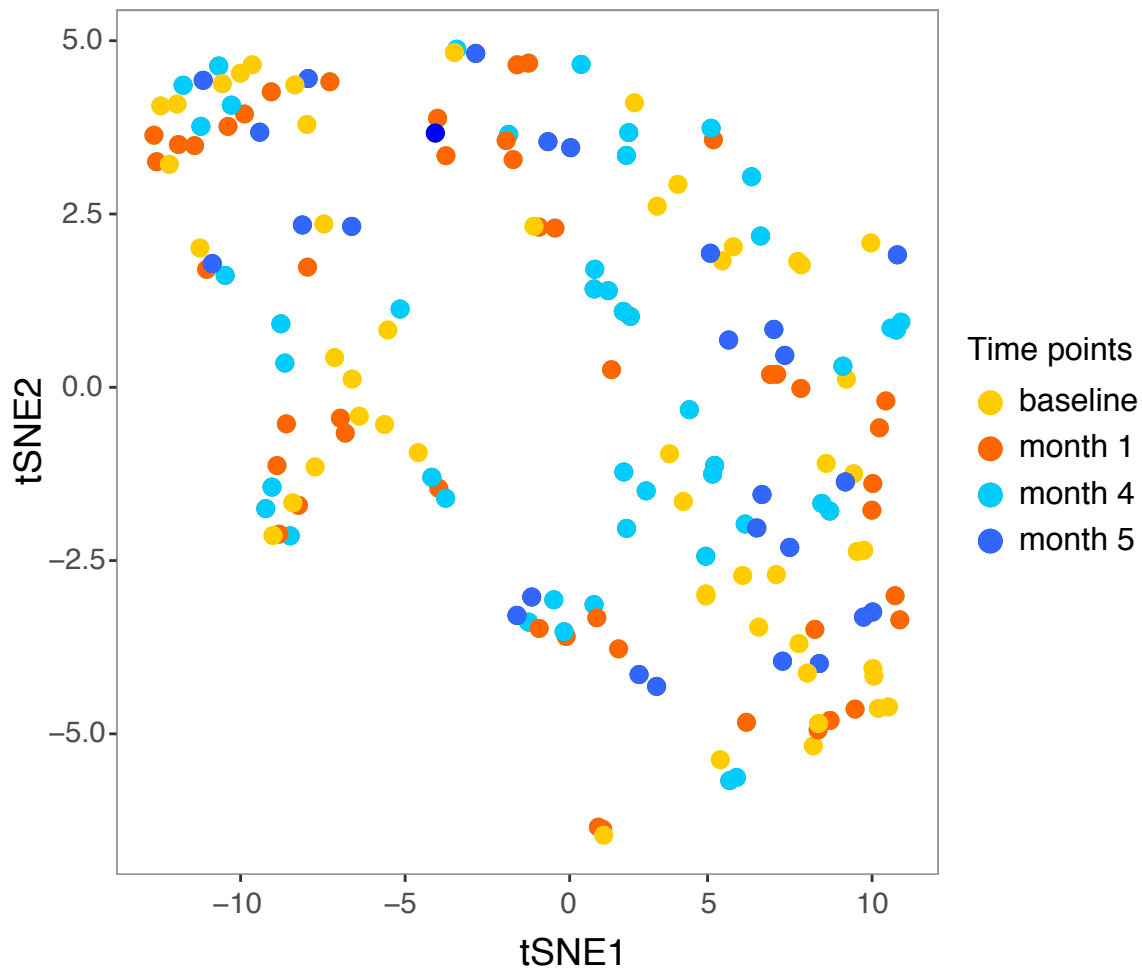

Supplement: Supplementary file 2 — Figure S2. tSNE of the lung microbiota, measured by Bray-Curtis distances. The different colors correspond to the different time points. Yellow: baseline, n = 52 samples; orange: month 1, n = 47; light blue: month 4, n = 46; dark blue: month 5, n = 27. (PDF 702 kb) [file 40168_2018_560_MOESM2_ESM.pdf]

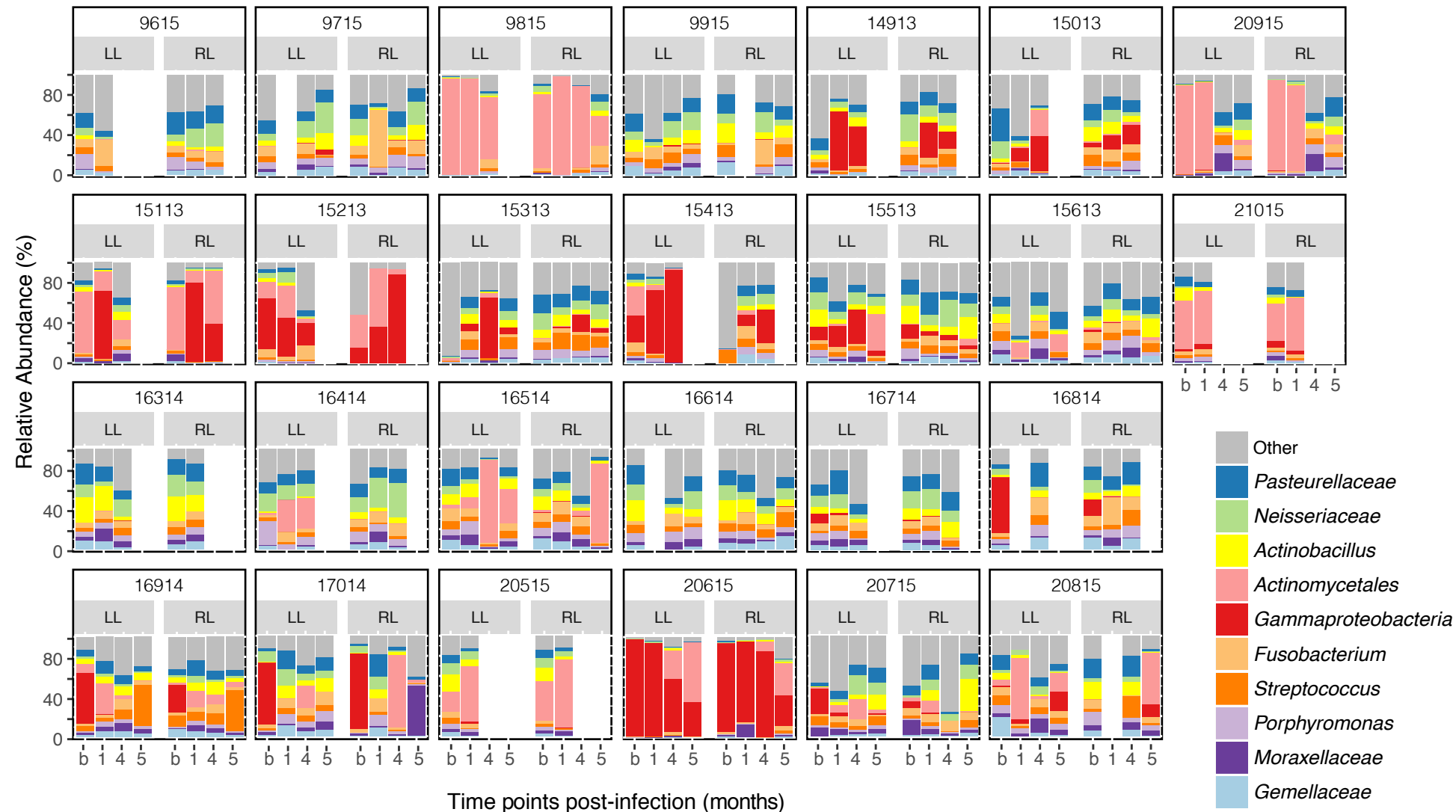

Supplement: Supplementary file 3 — Figure S3. Relative abundance of main taxa in the lung microbiota. For most monkeys, the microbial composition is relatively similar between right and left lobes within the same monkey, while the microbiota across monkeys is divergent. b: baseline; 1, 4, 5: months post-infection. LL: left lower lobe; RL: right lower lobe. (PDF 287 kb) [file 40168_2018_560_MOESM3_ESM.pdf]
